# In this month’s Bulletin

**DOI:** 10.2471/BLT.23.000423

**Published:** 2023-04-01

Viroj Tangcharoensathien et al. (230) call for submissions to an upcoming theme issue on global health and equity. Lia Tadesse et al. (231) introduce a World Health Assembly item on the strengthening of emergency, critical and operative care. 

Fid Thomson (234–235) reports on the need for a multisectoral response to cholera control in affected countries. Firdausi Qadri talks to Gary Humphreys (236–237) about the need for new cholera vaccines and increased vaccine production.

## Cambodia 

### Providing hepatitis C care

Daniel O’Keefe et al. (262–270) describe a pilot project initiated by nurses.

## China 

### Predicting cardiovascular disease risk 

Songchun Yang et al. (238–247) evaluate performance of the risk prediction model in ten regions. 

### How antibiotics are used 

Haishaerjiang Wushouer et al. (248–261) track trends in inpatient use.

### Can text messages help people to stop smoking?

Haoxiang Lin et al. (271–280) conduct a randomized controlled trial.

## India

### Children with community-acquired pneumonia

Shally Awasthi et al. (281–289) validate a tool that evaluates risk of death.

## Global

### Antibiotics: access, watch or reserve

Veronica Zanichelli et al. (290–296) present WHO’s approach to preventing antimicrobial resistance.

